# ﻿Two new species of *Diphya* Nicolet, 1849 (Araneae, Tetragnathidae) from Southwest China

**DOI:** 10.3897/zookeys.1124.86828

**Published:** 2022-10-12

**Authors:** Jianshuang Zhang, Qi Zhang, Feiyang Long, Hao Yu, Yin Yi

**Affiliations:** 1 The State Key Laboratory of Southwest Karst Mountain Biodiversity Conservation of Forestry Administration, School of life sciences, Guizhou Normal University, Guiyang, China; 2 The Key Laboratory of Plant Physiology and Development in Guizhou Province, School of life sciences, Guizhou Normal University, Guiyang, China; 3 School of Biological Sciences, Guizhou Education University, Guiyang, Guizhou, China

**Keywords:** DNA barcoding, identification key, morphology, taxonomy, tetragnathid

## Abstract

Two new species of tetragnathid spiders from Guizhou and Sichuang provinces of China are described: *Diphyaguiyang* J. Zhang & H. Yu, **sp. nov.** (♂♀) and *Diphyaweimiani* J. Zhang & H. Yu, **sp. nov.** (♀). Detailed descriptions, diagnoses, and photographs are provided for these two species, as well as a key and a distribution map for Chinese *Diphya* species. DNA barcodes (a partial fragment of the mitochondrial cytochrome oxidase subunit I gene, COI) of both new species were obtained for species delimitation, matching of different sexes, and future use in molecular studies.

## ﻿Introduction

*Diphya* Nicolet, 1849 is a small spider genus with an unusual distribution, it is disjunctively distributed in South America, southern Africa, and East Asia (Marusik et al. 2017; Omelko et al. 2020; World Spider Catalog 2022). *Diphya* currently includes 18 described species, with seven species recorded from Asia, six of which are known from China (Omelko et al. 2020; World Spider Catalog 2022).

The genus has been revised both regionally and on a worldwide scale (Tanikawa 1995; Álvarez-Padilla and Hormiga 2011; Marusik 2017; Omelko et al. 2020). However, debate is ongoing on the genus’s limit and subfamily placement (Álvarez-Padilla and Hormiga 2011; Marusik et al. 2017; Omelko et al. 2020). Marusik et al. (2017) have expressed doubts about the monophyly of the genus and thought that African, Asian, and South Neotropical species may in the future be considered to belong to separate genera. Despite of the dispute about the limits of this genus, most *Diphya* species have been well studied, especially several new species described in recent years. These species have been described in detail, alongside high-quality illustrations, to allow easy species recognition (Álvarez-Padilla and Hormiga 2011; Yu et al. 2014; Marusik 2017; Marusik and Omelko 2017a, b; Omelko et al. 2020).

While examining spiders recently collected from Guizhou and Sichuan provinces, southwestern China, we have found some *Diphya* specimens that belong to two undescribed species. With that, the total number of *Diphya* species in China reaches nine species, five known by both sexes. This makes China the country with the most *Diphya* species. The goal of this paper is to provide detailed descriptions, illustrations, and diagnosis of these two new species: *D.guiyang* J. Zhang & H. Yu, sp. nov. and *D.weimiani* J. Zhang & H. Yu, sp. nov. The DNA barcodes of these two species were obtained to confirm matching of the sexes (for *D.guiyang* sp. nov.) and future use in molecular studies. Additionally, an identification key and a distribution map for Chinese *Diphya* species are given.

## ﻿Materials and methods

Specimens in this study were collected by beating vegetation. The type specimens are deposited in the Museum of Guizhou Education University, Guiyang, China (MGEU; Hao Yu curator). Specimens were preserved in 95% alcohol and examined using an Olympus SZX7 stereomicroscope. Left male palps were examined and illustrated after dissection. Epigynes were removed and cleared in a warm 10% potassium hydroxide (KOH) solution. The vulvae were imaged after being embedded in Arabic gum. Images were captured with a Canon EOS 70D digital camera (20.2 megapixels) mounted on an Olympus CX41 compound microscope and assembled using Helicon Focus v. 6.80 image-stacking software. All measurements were obtained using an Olympus SZX7 stereomicroscope and are given in millimetres. Eye diameters were measured at the widest part. The total body length does not include the chelicerae or spinnerets. Leg lengths are given as total length (femur, patella+tibia, metatarsus, tarsus). The terminology used in the text and figure legends follows Marusik (2017), Marusik et al. (2017), and Omelko et al. (2020).

The abbreviations used in the text are: **A** = atrium; **AER** = anterior eye row; **ALE** = anterior lateral eye; **AME** = anterior median eye; **C** = conductor; **Cd** = copulatory duct; **Co** = copulatory opening; **Cy** = cymbium; **Dp** = dorsal process; **Em B** = basal portion of embolus; **Em T** = terminal portion of embolus; **Fd** = fertilisation duct; **Ip** = intermediate process; **Lp** = lateral pocket; **MOQ** = median ocular quadrangle; **MOQA** = MOQ anterior width; **MOQL** = length of MOQ; **MOQP** = MOQ posterior width; **Pc** = paracymbium; **PLE** = posterior lateral eye; **PME** = posterior median eye; **RER** = posterior eye row; **R** = receptacle; **Ra** = anterior chamber of receptacle; **Rp** = posterior chamber of receptacle; **Sb** = septal base; **Se** = septum; **Ss** = septal stem; **St** = subtegulum; **Te** = tegulum; **Vp** = ventral process.

The distribution map was generated with ArcGIS v. 10.5 (Environmental Systems Research Institute, Inc.). Due to lack of locality coordinates in previous publications, locality coordinates for all known species are derived from ArcGIS, except for *D.qianica* and *D.tanasevitchi*, which were copied from the original publications (see Zhu et al. 2003: 57; Zhang et al. 2003: 407).

To obtain the DNA barcodes, a partial fragment of the mitochondrial cytochrome oxidase subunit I (COI) gene was amplified and sequenced for four specimens, using the primers LCO1490 (5′-GGTCAACAAATCATCATAAA-GATATTGG-3′) and C1-N-2776 (5′-GGATAATCAGAATANCGNCGAGG-3′). For additional information on extraction, amplification, and sequencing procedures, see Wheeler et al. (2016). Sequences were trimmed to 651 bp. All sequences were analysed using BLAST and are deposited in GenBank. The accession numbers are provided in Table 1.

**Table 1.** Voucher specimen information (sequence length 651bp).

Speices	Voucher code	Sex	GenBank accession number
*D.guiyang* sp. nov.	MGEU-TET-21-001 (YHTET001)	♂	OP476467
	MGEU-TET-21-002 (YHTET002)	♀	OP476466
	MGEU-TET-21-003 (YHTET003)	♂	OP476465
*D.weimiani* sp. nov.	MGEU-TET-22-001 (YHTET004)	♀	OP476468

## ﻿Taxonomy

**Family Tetragnathidae Menge, 1866**

### ﻿Subfamily Diphyainae Simon, 1894

#### ﻿Genus Diphya

Taxon classificationAnimaliaAraneaeTetragnathidae

Nicolet, 1849

F37A91E7-FBF3-5F3B-8CB8-F6FCFB866F6C

##### Type species.

*Diphyamacrophthalma* Nicolet, 1849.

##### Diagnosis.

For details see Álvarez-Padilla and Hormiga (2011) and Marusik et al. (2017).

##### Description.

The genus is well described by Tanikawa (1995) and Álvarez-Padilla and Hormiga (2011).

##### Composition and distribution.

For details see WSC (2022).

##### Comments.

Although the debate on the limit of this genus remains open, a review of the genus *Diphya* is not within the scope of this work. Consequently, the present study follows WSC (2022) and Omelko et al. (2020) and temporarily places both new species in *Diphya* sensu lato for the lack of a better solution.

### ﻿Key to *Diphya* species occurring in China

**Table:** 

1	Males	**2**
–	Females	**5**
2	Paracymbium simple and unbranched; embolus slender, distinctly longer than tegulum width, whip-shaped	**3**
–	Paracymbium complex, with at least 3 processes (or outgrowths); embolus short and stout, shorter than tegulum width, embolar tip C-shaped, laminar or blade-shaped (Fig. 1A, B, D)	**4**
3	Paracymbium thumb-like, slightly longer than wide; the middle part of embolus close to tegulum	***D.okumae***
–	Paracymbium distinctly longer than wide, >-shaped; the middle part of embolus well separated from tegulum	***D.tanasevitchi***
4	Paracymbium with 4 processes; embolar tip C-shaped, thick and heavily sclerotized, apex relatively sharp	***D.wulingensis***
–	Paracymbium with 3 processes (Fig. 1A, B, D); embolar tip blade-shape, hyaline, apex relatively wide (Fig. 2B–E)	***D.guiyang* sp. nov.**
5	Epigynal atrium (or called fovea) distinct, lack of septum (Fig. 5A, C, E)	**6**
–	Epigynal atrium indistinct, divided or covered by septum (Fig. 3A, C, E)	**7**
6	Epigynal atrium located at anterior part of epigynal plate; copulatory ducts short and simple, not longer than epigyne length, not convoluted	***D.okumae***
–	Epigynal atrium located posteriorly; copulatory ducts long, longer than epigyne length, strongly convoluted (Fig. 5A–E)	***D.weimiani* sp. nov.**
7	Septal stem narrow, less than 1/2 of septal base	**8**
–	Septal stem relatively wide, not less than 1/2 of septal base (Fig. 3A, C, E)	**10**
8	Receptacles not subdivided	***D.tanasevitchi***
–	Receptacles subdivided in 2 chambers	**9**
9	Receptacles separated by 4 diameters	***D.wulingensis***
–	Receptacles separated by no more than 1 diameter	***D.qianica***
10	Epigynal plate anteriorly with a V-shaped depression, septal base narrower than septal stem	***D.songi***
–	Epigynal plate anteriorly without depression, septal base wider than septal stem (Fig. 3A, C, E)	**11**
11	Septum T-shaped, with a wide head (anterior part of septum); septal base short, about 1/3 of septum length (Fig. 3A, C, E); abdomen dorsally with 5 pairs of irregularly shaped black marks (Fig. 4D)	***D.guiyang* sp. nov.**
–	Septum shaped like outline of a vase, lack head; septal base large, about 4/5 of septum length abdomen dorsally only with 2 pairs of muscular depressions	***D.taiwanica***

#### ﻿ Diphya guiyang

Taxon classificationAnimaliaAraneaeTetragnathidae

J. Zhang & H. Yu sp. nov.

CD06A933-472A-54D8-8D40-4BEA880BB212

https://zoobank.org/D5FB012F-6152-4ADC-8619-1A3F2B874600

**Figure 1.**
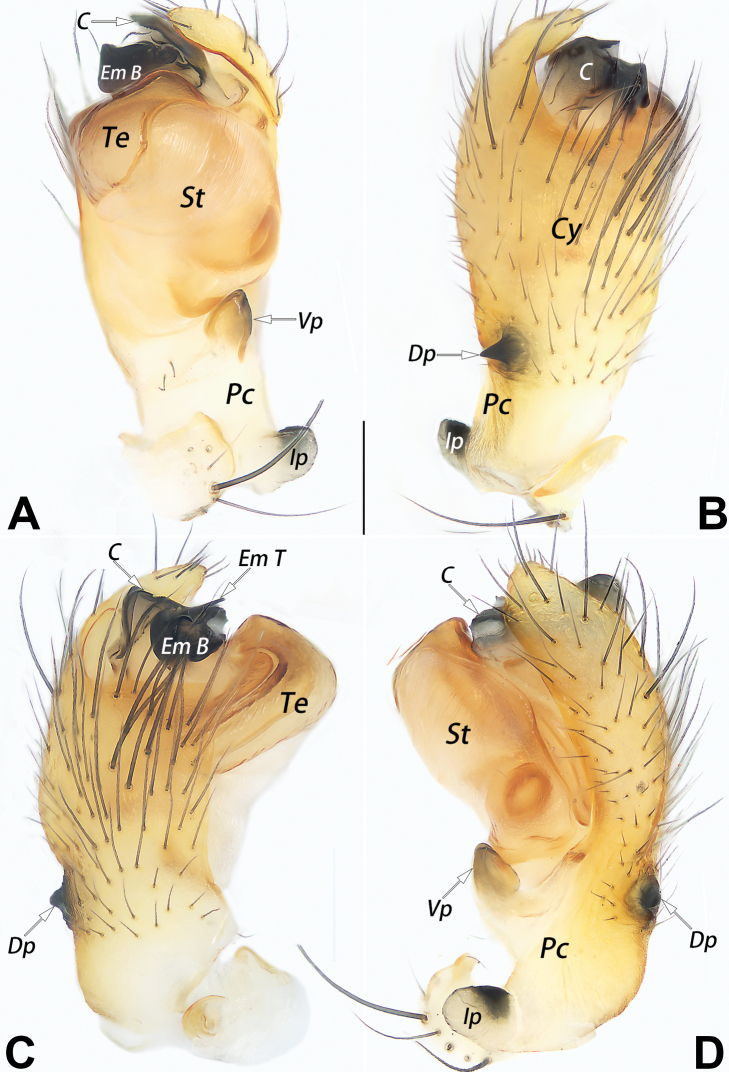
Male palp of the holotype of *Diphyaguiyang* sp. nov. **A** ventral view **B** dorsal view **C** prolateral view **D** retrolateral view. Abbreviations: C = conductor; Cy = cymbium; Dp = dorsal process; Em B = basal portion of embolus; Em T = terminal portion of embolus; Ip = intermediate process; Pc = paracymbium; St = subtegulum; Te = tegulum; Vp = ventral process. Scale bars: 0.2 mm.

**Figure 2.**
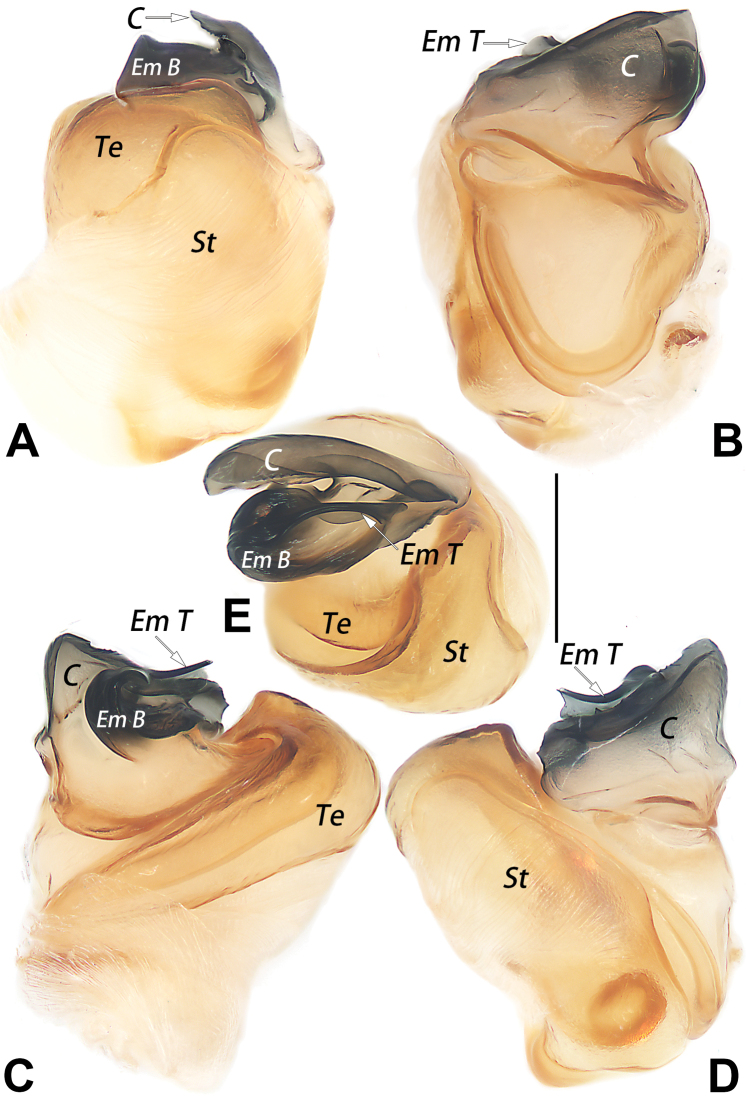
Male palpal bulb of the holotype of *Diphyaguiyang* sp. nov. **A** ventral view **B** dorsal view **C** prolateral view **D** retrolateral view **E** anterior view. Abbreviations: C = conductor; Em B = basal portion of embolus; Em T = terminal portion of embolus; St = subtegulum; Te = tegulum. Scale bars: 0.2 mm.

**Figure 3.**
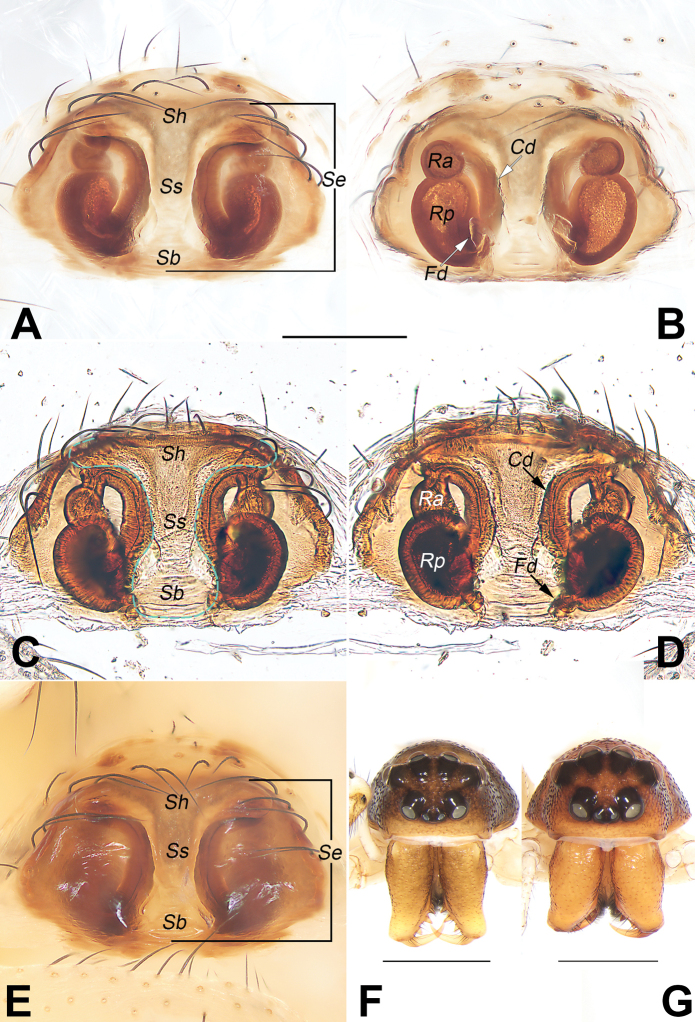
*Diphyaguiyang* sp. nov. **A–E** female paratype and male holotype, epigyne **A, B** macerated epigyne, ventral and dorsal **C, D** epigyne, macerated and embedded in Arabic gum, ventral and dorsal **E** intact epigyne **F, G** ventral view frontal view of prosoma **F** male **G** female. Abbreviations: Cd = copulatory duct; Fd = fertilisation duct; Ra = anterior chamber of receptacle; Rp = posterior chamber of receptacle; Sb = septal base; Se = septum (dashed line in C showing margin of septum); Sh = septal head; Ss = septal stem. Scale bars: 0.2 mm (**A–E**); 1 mm (**F, G**).

**Figure 4.**
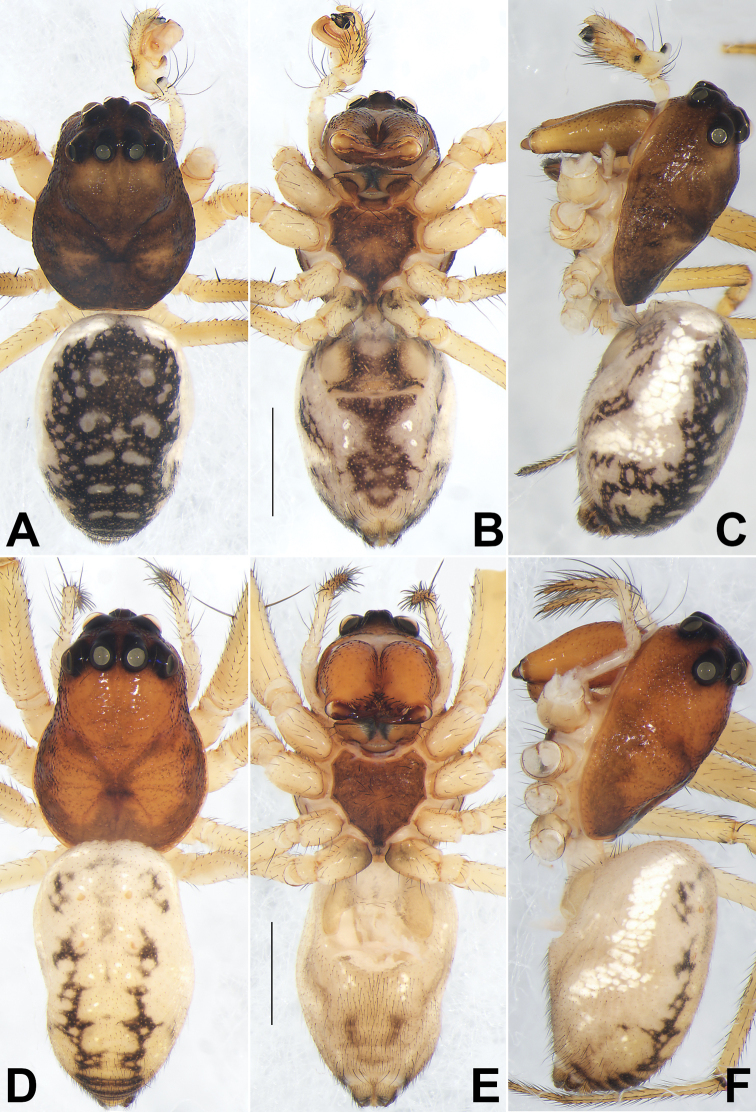
*Diphyaguiyang* sp. nov. **A–C** habitus of the male holotype **D–F** female paratype **A, D** dorsal view **B, E** ventral view **C, F** lateral view. Scale bars: 1 mm.

Figs 1 , 2 , 3 , 4 , 7

##### Material examined.

***Holotype*.** ♂ (MGEU-TET-21-001, YHTET001), China: Guizhou Province: Guiyang City: Nanming District, Guiyang Forest Park, 26.55°N, 106.75°E, ca 1165 m, 10 August 2021, H. Yu et al. leg., hand picking on shrubs. ***Paratypes***: 1♂ 1♀ (MGEU-TET-21-002–003, YHTET002–003), same data as holotype.

##### Other material examined.

1♂ 2♀, same data as holotype.

##### Diagnosis.

The male of *D.guiyang* sp. nov. resembles that of *D.wulingensis* Yu, Zhang & Omelko, 2014 in having a similar complex paracymbium with several processes (other species have simple unbranched paracymbium and cymbial process) but can be distinguished from it by the different shape, locations, and number of paracymbial processes and by the different shape and degree of sclerotization of the embolus. In *D.guiyang* sp. nov., the paracymbium has 3 processes (vs 4), the intermediate process (Ip) is thumb-like and originates from the distal end of the paracymbium, close to tibia (Fig. 1A, B, D) (vs papilliform and located at the proximal margin of the paracymbium, well-separated from tibia); the embolar tip (Em T) is blade-shaped, hyaline, and with a relatively wide apex (Fig. 2B–E). (vs C-shaped, thick, heavily sclerotized, and with the apex relatively sharp; Yu et al. 2014: 31, figs 5, 10, 12; Marusik et al. 2017: 143, figs 13–15, 17). The female of *D.guiyang* sp. nov. also resembles that of *D.wulingensis* in having a similarly shaped vulva, but can be separated by having the septal base (Sb) relatively narrow (less than 1/3 of the epigynal plate width) (vs wide, about ½ of the plate width) (cf. Fig. 3A, C, E and Marusik et al. 2017: 143, figs 10, 11), and by the kidney-shaped posterior chamber of receptacle (Rp), which is distinctly larger than the anterior chamber (Ap) (vs both Ap and Rp nearly globular and Ap slightly larger than Rp) (cf. Fig. 3B, D and Yu et al. 2014: 31, figs 4, 9). In addition, the two species can be reliably separated by the abdominal pattern: dorsum of the abdomen centrally with a distinct symmetrical pattern in *D.guiyang* sp. nov. (Fig. 4A, D), vs without pattern centrally and only with black marks on both sides (Yu et al. 2014: 31, figs 1, 2; Marusik et al. 2017: 143, figs 1, 2, 5).

##### Etymology.

The species name is derived from the type locality; noun in apposition.

##### Description.

**Male.** Holotype (Figs 3F, 4A–C): total length 4.18; carapace 2.04 long, 1.48 wide; abdomen 2.14 long, 1.46 wide. ***Carapace*** dark brown, slightly lighter between PER and cervical groove. Clypeus dark brown, distinctly higher than AME diameter. Eye sizes and interdistances: AME 0.07, ALE 0.14, PME 0.15, PLE 0.14, AME–AME 0.09, AME–ALE 0.08, PME–PME 0.16, PME–PLE 0.20, MOQL 0.66, MOQA 0.22, MOQP 0.48. Chelicerae light brown, with 3 promarginal and 4 retromarginal teeth. Sternum coloured the same as carapace, 0.76 long, 0.85 wide.

***Abdomen*** dorsally dark with 5 pairs of spots (anterior pair circular, 2^nd^ pair comma-shaped and largest, posterior 3 pairs represented by 6 short transverse bands), surrounded by line consisting of small white spots. Lateral sides whitish. Ventrally with irregularly shaped black pattern.

***Legs*** uniformly yellowish. Leg measurements: I 8.90 (2.31, 2.62, 2.80, 1.17), II 7.56 (2.12, 2.30, 2.19, 0.95), III 6.70 (1.99, 2.06, 1.78, 0.87), IV – (1.87, 1.83, –, –).

***Palp*** (Figs 1A–D, 2A–E): paracymbium (Pc) complex, with 3 processes: both ventral process (Vp) and intermediate process (Ip) large, thumb-like, dorsal process (Dp) relatively small, tooth-shaped; Vp originating from 1/3 to 1/4 proximal part of cymbium, slightly curved, apex pointing distally; Ip originating from base of cymbium, apex pointing ventrally; Dp originating from ca 2/5 proximal part of cymbium, slightly curved, apex pointing retrolaterlly. Cymbium concave prolaterally. Subtegulum (St) large, hiding tegulum in retrolateral view; tegulum (Te) circular; sperm duct indistinct in ventral view. Conductor (C) laminar and hyaline, slightly smaller than tegulum, originating from dorsal-retrolateral portion of tegulum. Embolus (Em) slightly shorter than conductor, twisted around axis; embolar base (Em B) relatively sclerotized; embolar tip (Em T) blade-shaped, apex as wide as Em B and pointing ventrally.

**Female** (paraype: MGEU-TET-21-002) (Figs 3G, 4D–F). Total length 4.48; carapace 1.99 long, 1.53 wide; abdomen 2.49 long, 1.55 wide. ***Carapace*** uniformly red-brown, cervical groove and radial grooves distinct. Clypeus orange, distinctly higher than AME diameter. Eye sizes and interdistances: AME 0.09, ALE 0.24, PME 0.26, PLE 0.19; AME–AME 0.09, AME–ALE 0.08, PME–PME 0.08, PME–PLE 0.06. MOQL 0.81, MOQA 0.24, MOQP 0.58. Chelicerae light orange, with 3 promarginal and 4 retromarginal teeth. Sternum 0.88 long, 0.87 wide, slightly darker than carapace. ***Abdomen*** basically yellowish white, dorsum centrally with indistinct, broken lengthwise band, reaching posterior half; with 2 pairs of muscular depressions located at two sides of lengthwise band; with 5 pairs of irregularly shaped black marks (frontal pair of marks largest), running longitudinally extending ca 4/5 of abdomen length. Lateral sides whitish. Ventrally yellowish white, without distinct pattern.

***Legs*** uniformly yellowish. Measurements of legs: I 8.06 (2.11, 2.60 2.25, 1.10), II 7.22 (2.09, 2.24, 1.93, 0.96), III 4.62 (1.42, 1.44, 1.14, 0.62), IV 6.15 (1.93, 1.99, 1.56, 0.67).

***Epigyne*** (Fig. 3A–E). Plate distinctly wider than long. Septum (Se) T-shaped, consisting of a transverse head (Sh), a narrow stem (Ss) and nose-shaped base (Sb); septal head wide, about 2/3 of the epigynal plate width; septal stem (Ss) slightly narrower than septal base, about twice longer that septal base length; septal base (Sb) shaped like a nose, nearly as wide as long. Copulatory openings indistinct, located in rebordered groove of lateral margins of septum. Copulatory ducts (Cd) diverging posteriorly, running along with lateral margin of septum. Receptacle subdivided in 2 chambers; anterior chamber (Ra) globular, relatively small, widely separated by ca 2.7 diameters; posterior chamber (Rp) kidney-shaped, distinctly larger than anterior chamber, 1.5 times longer than wide, separated by ca 1.3 widths. Fertilization ducts (Fd) acicular, membranous, located on posterior-interlateral surface of Rp.

##### Distribution.

Known only from the Guiyang City, Guizhou Province, China (Fig. 7).

#### ﻿ Diphya weimiani

Taxon classificationAnimaliaAraneaeTetragnathidae

J. Zhang & H. Yu sp. nov.

5A974D54-5188-5A0D-B590-7182CDDC5372

https://zoobank.org/F79B3587-2128-4D61-BA1F-9AD6BA99A094

Figs 5 , 6 , 7

##### Material examined.

***Holotype*.** ♀ (MGEU-TET-22-001, YHTET004), CHINA: Sichuan Province: Yaan City: Lushan County, Longmen Town, Longmen Mountain, 30.23°N, 103.02°E, ca 885 m, 14 May 2022, M. Wei leg. ***Paratype***: 1♀ (MGEU-TET-22-002), Guizhou Province: Qiandongnan Miao and Dong Autonomous Prefecture: Leishan County, Leigong Mountain, 26.38°N, 108.20°E, ca 1965 m, 27 July 2021, Y.C. Lin and M. Wei leg.

##### Diagnosis.

**Figure 5.**
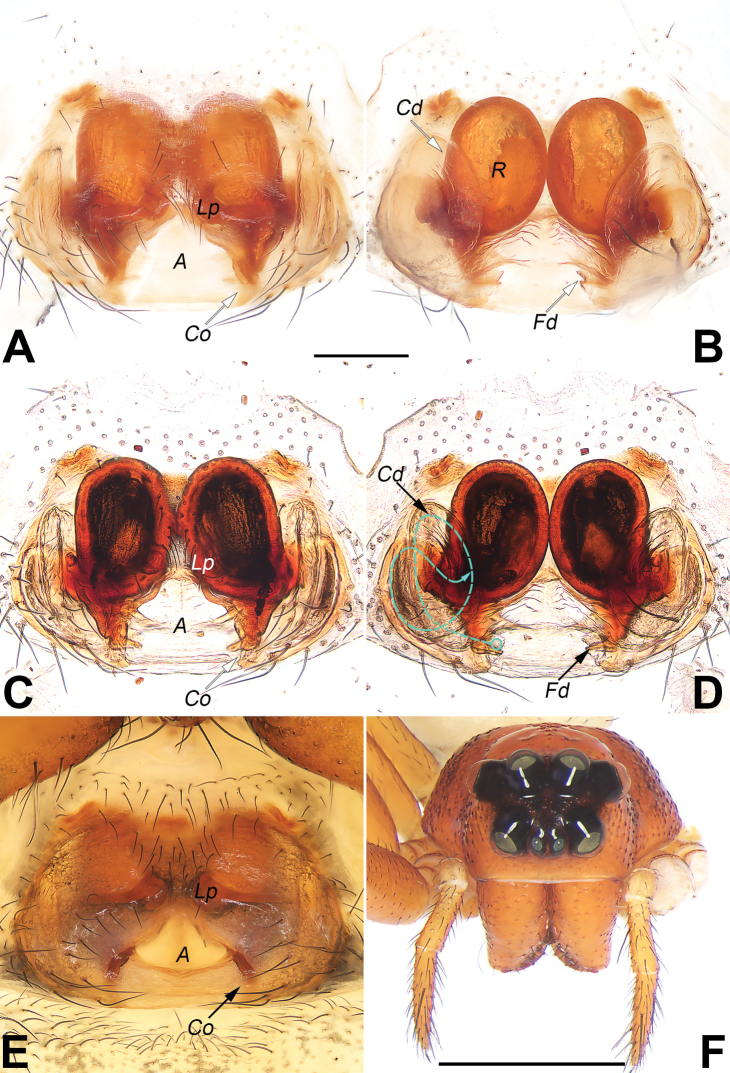
*Diphyaweimiani* sp. nov., female holotype, epigyne (**A–E**) and frontal view of prosoma (**F**). **A, B** macerated epigyne, ventral and dorsal **C, D** epigyne, macerated and embedded in Arabic gum, ventral and dorsal **E** intact epigyne, ventral view **F** female. Abbreviations: A = atrium; Cd = copulatory duct (dashed line in Fig. 5D showing schematic course of copulatory duct, dorsal); Co = copulatory opening; Fd = fertilisation duct; Lp = lateral pocket; R = receptacle. Scale bars: 0.2 mm (**A–E**); 1 mm (**F**).

The new species is easily distinguished from other congeners except *D.albula* (Paik, 1983) (Seo 2005: 49, figs 1, 2), *D.macrophthalma* Nicolet, 1849 (Marusik and Omelko 2017b: 25, 26, 30), and *D.okumae* Tanikawa, 1995 (Tanikawa 1995: 102, fig. 12; Zhu et al. 2003: 56, fig. 22) by the atrium distinct, and by lack of septum (vs atrium indistinct, divided or covered by septum; septum with variable shapes but distinct in all other *Diphya* species, such as *D.guiyang* sp. nov.; Fig. 3A, C, E), but differ from the latter three by the atrium located posteriorly (Fig. 5A, C, E) (vs located anteriorly), the copulatory ducts strongly entwined (Fig. 5B, D) (vs not entwined), and by the receptacles not subdivided (Fig. 5B, D) (vs receptacles subdivided in 2 chambers).

##### Etymology.

The specific name is a patronym after Mian Wei (Chengdu City, China), the collector of the type material.

##### Description.

**Figure 6.**
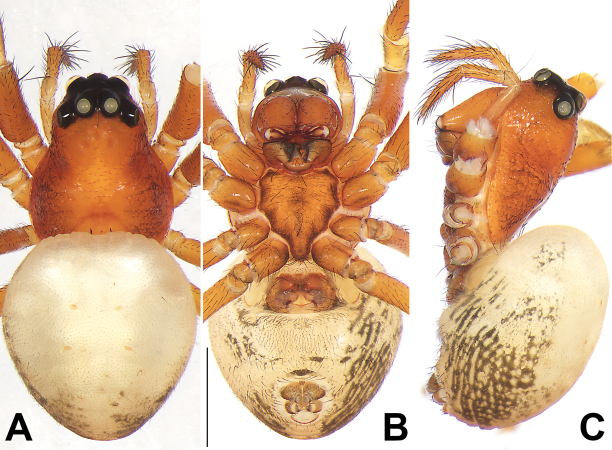
Habitus of the female holotype of *Diphyaweimiani* sp. nov. **A** dorsal view **B** ventral view **C** lateral view. Scale bars: 1 mm.

**Female.** Holotype (Figs 5F, 6A–C): total length 3.79; carapace 1.57 long, 1.55 wide; abdomen 2.22 long, 1.55 wide.

***Carapace*** red-brown, marginally slightly darker. Clypeus light orange, distinctly higher than AME diameter. Eye sizes and interdistances: AME 0.08, ALE 0.16, PME 0.15, PLE 0.16, AME–AME 0.06, AME–ALE 0.07, PME–PME 0.12, PME–PLE 0.16, MOQL 0.57, MOQA 0.20, MOQP 0.45. Chelicerae light orange, with 3 promarginal and 4 retromarginal teeth. Sternum coloured as carapace, 0.80 long, 0.68 wide.

***Abdomen*** dorsally uniformly yellowish white, dorsum with two pairs of inconspicuous muscle depressions; laterally with lengthwise reticular pattern; ventrally white with no distinct pattern.

***Legs*** uniformly red-brown. Leg measurements: I 5.58 (1.47, 1.85, 1.46, 0.80), II 5.10 (1.43, 1.65, 1.31, 0.71), III 3.36 (1.03, 1.03, 0.81, 0.49), IV 1.38 (1.44, 1.27, 1.14, 0.53).

***Epigyne*** (Fig. 5A–E). Plate distinctly wider than long, with an atrium located posteriorly, receptacles and copulatory ducts indistinctly visible through integument. Atrium (A) shaped like an equilateral triangle, with rebordered margin, about 1/2 epigyne length and 1/3 epigyne width. Lateral pocket (Lp) located anteriorly to atrium, more or less comma-shaped, heavily sclerotized. Copulatory openings (Co) indistinct, located at basolateral atrial borders. Copulatory ducts (Cd) strongly entwined, loop twice before connecting to receptacles. Receptacles (R) oval or balloon-shaped, not subdivided, relatively large, ca 1.3 times longer than wide, surface smooth; two receptacles close together. Fertilization ducts (Fd) acicular, membranous, located on posterior surface of receptacles.

**Male.** Unknown.

##### Comments.

According to WSC (2022), only two species of *Diphya* are known only from males: *D.bicolor* Vellard, 1926 from Brazil, and *D.leroyorum* Omelko, Marusik & Lyle, 2020 from South Africa. However, neither could be matched with *D.weimiani* sp. nov. due to the long distance between their type localities (China is tens of thousands of kilometres from Brazil and South Africa).

##### Distribution.

**Figure 7.**
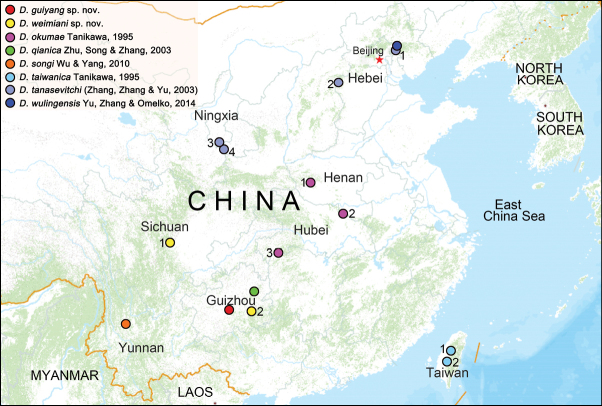
Distribution records of the *Diphya* species in China. *D.guiyang* sp. nov. (scarlet circle: Guizhou Province, Guiyan City), *D.weimiani* sp. nov. (yellow circle: 1. Sichuan Province, Mount Longmen; 2. Guizhou Province, Mount Leigong), *D.okumae* Tanikawa, 1995 (carmine circle: 1. Hennan Province, Xinyang City; 2. Hennan Province, Neixiang County; 3. Hubei Province, Hefeng County), *D.qianica* Zhu, Song & Zhang, 2003 (greent circle: Guizhou Province, Mount Fanjing), *D.songi* Wu & Yang, 2010 (orange circle: Yunnan Province, Mount Canshan), *D.taiwanica* Tanikawa, 1995 (light blue circle: 1. Taiwan Province, Mount Pahsien-shan; 2. Taiwan Province, Mount Alishan); *D.tanasevitchi* (Zhang, Zhang & Yu, 2003) (lilac circle: 1. Hebei Province, Mount Wuling; 2. Hebei Province, Pingshan County; 3. Ningxia Hui Autonomous Region, Delong County; 4. Ningxia Hui Autonomous Region, Jingyuan County), *D.wulingensis* Yu, Zhang & Omelko, 2014 (dark blue circle: Hebei Province, Mount Wuling).

Known from the Mount Longmen Mountain (Sichuan Province), and Mount Leigong Mountain (Guizhou Province), China (Fig. 7).

## Supplementary Material

XML Treatment for Diphya

XML Treatment for Diphya guiyang

XML Treatment for Diphya weimiani
